# Novel Multiplex PCR Assay and Its Application in Detecting Prevalence and Antibiotic Susceptibility of Porcine Respiratory Bacterial Pathogens in Guangxi, China

**DOI:** 10.1128/spectrum.03971-22

**Published:** 2023-03-14

**Authors:** Jing Rao, Xinchen Wei, Huan Li, Zhewei Zhang, Jiahui Liu, Mengjie Lian, Weiwei Cao, Long Yuan, Beibei Dou, Yanhong Tian, Huanchun Chen, Jinquan Li, Weicheng Bei

**Affiliations:** a State Key Laboratory of Agricultural Microbiology, Hubei Hongshan Laboratory, College of Veterinary Medicine, Huazhong Agricultural University, Wuhan, China; b Cooperative Innovation Center for Sustainable Pig Production, Wuhan, China; c Hubei Key Laboratory of Agricultural Bioinformatics, College of Informatics, Huazhong Agricultural University, Wuhan, China; Changchun Veterinary Research Institute

**Keywords:** PRDC, porcine respiratory bacterial pathogens, multiplex PCR, epidemiology, antibiotic resistance, Guangxi Province

## Abstract

Porcine respiratory disease complex (PRDC) is a serious disease caused by multiple pathogens which inflicts huge economic losses on the pig industry. Investigating the epidemiology of porcine respiratory bacterial pathogens (PRBPs) in specific geographic areas and exploring the antibiotic susceptibility of local strains will contribute to the prevention and control of PRDC. However, the epidemiology of PRBPs in Guangxi Province remains unclear, and existing diagnostic methods have multiple limitations, such as high costs and the detection of only a single pathogen at a time. In this study, we developed a multiplex PCR assay for Streptococcus suis, Glaesserella parasuis, Actinobacillus pleuropneumoniae, Pasteurella multocida, and Mycoplasma hyopneumoniae, and investigated the prevalence of PRBPs in pigs with respiratory symptoms in Guangxi Province. The isolates from positive samples were subjected to susceptibility tests to 16 antibiotics. Our results indicated that of the 664 samples from pigs with respiratory symptoms, 433 (65.21%), 320 (48.19%), 282 (42.47%), 23 (3.46%), and 9 (1.36%), respectively, carried each of these 5 pathogens; 533 samples were positive; and 377 (56.78%) carried multiple pathogens simultaneously. The dominant PRBPs in pigs with respiratory symptoms in Guangxi province were S. suis, G. parasuis, and A. pleuropneumoniae, which frequently co-infected swine herds. Most of the isolates (A. pleuropneumoniae, G. parasuis, S. suis, and P. multocida) were sensitive to cefquinome, ceftiofur, trimethoprim-sulfamethoxazole (TMP-SMX), and tiamulin antibiotics. We developed a rapid specific multiplex PCR assay for PRBPs. Our findings provide new information on the epidemiology of PRBPs in Guangxi Province and offer a reference for developing drug targets against PRDC.

**IMPORTANCE** Pigs are closely associated with humans as the most common food animals and the vectors of numerous pathogens. PRDC, caused by multiple pathogens, is a serious disease that can cause growth retardation in swine and even sudden death. Due to the droplet transmission of PRBP and the similar clinical signs of different pathogen infections, most pig farms struggle to identify and control PRBPs, leading to the abuse of antibiotics. In addition, some PRBPs have the potential to infect humans and threaten human health. Therefore, this study developed a multiplex PCR method targeting PRBPs, investigated the prevalence of these pathogens, and tested their antibiotic susceptibility. Our studies have important implications for public health safety and the development of the pig industry.

## INTRODUCTION

Porcine respiratory disease complex (PRDC) is the most common disease in pig farming; it causes growth retardation, poor feed conversion, and severe acute mortality, posing a very serious threat to the global pig industry (1). PRDC is frequently caused by multiple pathogenic microorganisms, involving several viral pathogens such as porcine reproductive and respiratory syndrome virus (PRRSV), porcine circovirus type 2 (PCV2), and swine influenza virus (SIV), and multiple bacterial pathogens such as Actinobacillus pleuropneumoniae, Glaesserella parasuis, Streptococcus suis, Pasteurella multocida, and Mycoplasma hyopneumoniae (2–9). The pathogens that cause PRDC are usually classified as primary and secondary pathogens. When infecting an organism, primary pathogens tend to disrupt the host defense barrier and provide the necessary conditions for secondary pathogens to infect. Each pathogen can cause PRDC independently, and the clinical signs caused by different pathogen infections are very similar. Simultaneous infection with multiple pathogens can cause more severe symptoms and lesions and even result in higher mortality than single-pathogen infections (2–6). Viral pathogens are frequently considered the main factors which cause PRDC, but numerous bacteria also play important roles in the development of PRDC (9–14). Porcine respiratory bacterial pathogen (PRBP) infection can decrease the resistance of swine, aggravating the progression of disease or even causing sudden death (10, 15, 16). In addition, S. suis and P. multocida have the potential to infect humans and threaten human health. PRDC has become a serious problem which urgently needs to be solved in large-scale Chinese pig farms (17–21). However, the prevalence of PRBPs in Guangxi Province, where several large-scale pig farms are located, remains largely unknown.

Due to the droplet transmission of PRBPs and the similar clinical signs of different pathogen infections, most pig farms struggle to identify and control PRBPs (15, 18, 20, 22). In pig farms where PRBPs are epidemic, broad-spectrum antibiotics are frequently employed as the only treatment; however, this untargeted blind medication may increase costs and cause a vicious circle of antibiotics and resistant pathogens, dramatically damaging the economic benefits of pig farms (5, 18, 22).

Usually, PRBPs are identified by species-specific methods, and the specific serovar is further identified by serotype-specific methods. Numerous methods such as PCR, LAMP (loop-mediated isothermal amplification), and CRISPR-Cas systems have been established to identify PRBPs; however, multiple factors involving testing costs, operation techniques, and the limitation of testing for only a single pathogen at a time limit large-scale application of these methods (23–26). Rapid and accurate methods for the identification of multiple pathogenic bacteria through a single reaction are urgently needed, and they are of great significance for the prevention and control of PRDC.

**FIG 1:**
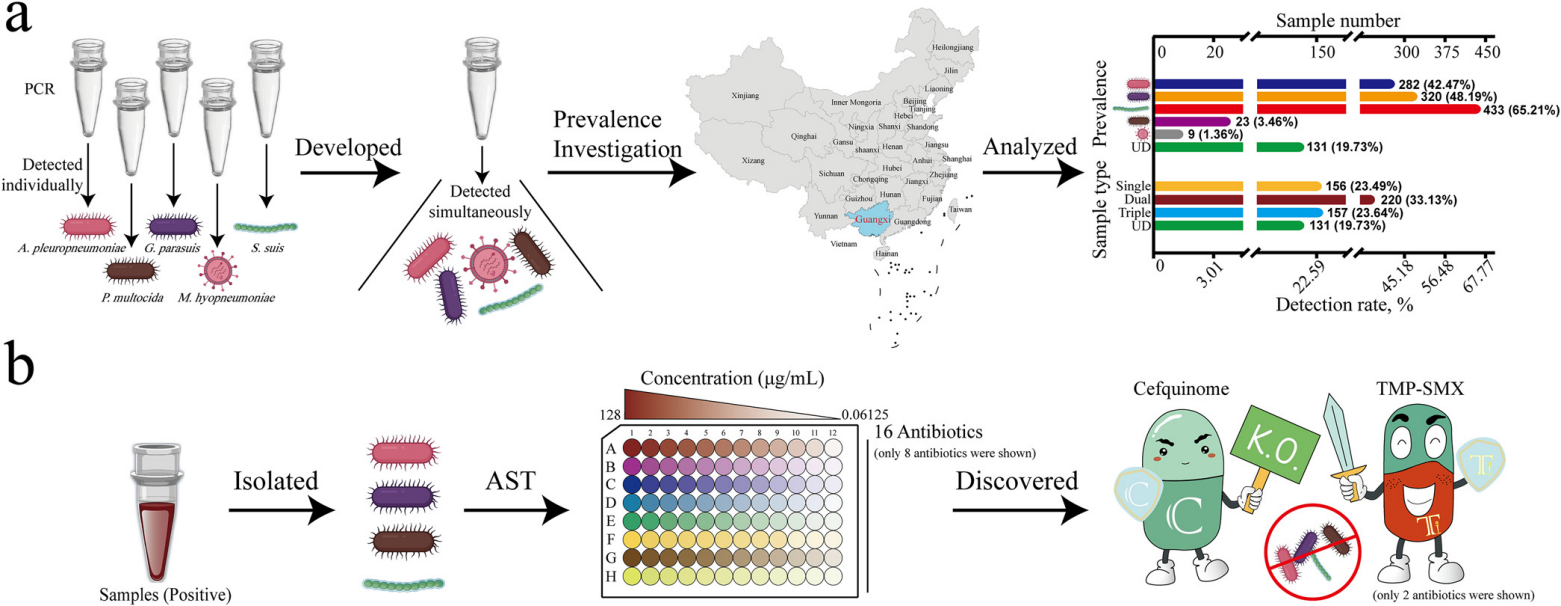
Schematic of studies on porcine respiratory bacterial pathogens (PRBPs) circulating in Guangxi Province. (a) Establishment and application of multiplex PCR method for PRBPs in Guangxi Province. (b) Antibiotic susceptibility testing (AST) of isolates from positive samples. The source file of the map in panel a was provided by Huanzhong Agricultural University and was modified and exported in ArcGIS.

Therefore, in the present study, we developed a multiplex PCR method for detecting common PRBPs, including A. pleuropneumoniae, *G. parasuis*, S. suis, P. multocida, and M. hyopneumoniae, investigated the prevalence of these pathogens, and tested the antibiotic susceptibility of isolates (Fig. 1). The results of this study will reveal the epidemiological characteristics of PRBPs in Guangxi Province and provide a reference for the development of clinical therapeutic drugs. Our work contributes to understanding of the distribution of PRBPs in China and complements the global PRBP prevalence database. Our research has important implications for public health and the development of the pig industry.

## RESULTS

### Development of multiplex PCR assay method.

**FIG 2:**
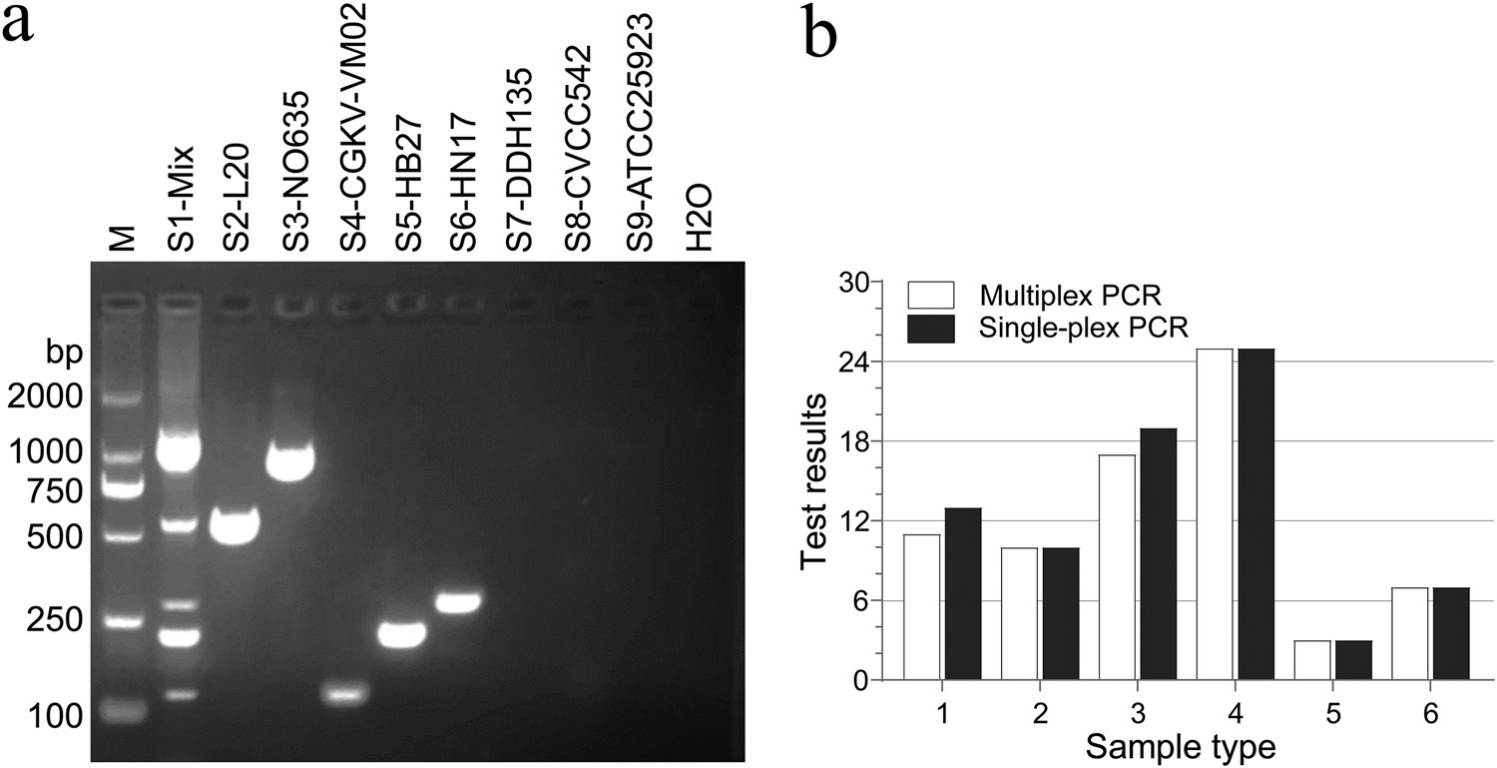
Evaluations on multiplex PCR. (a) Specificity evaluation of multiplex PCR. Lane M, DL2000 DNA molecular weight marker; lane S1, extracted genomic DNA mixture of reference strain (Table 4); lane S2, Actinobacillus pleuropneumoniae L20; lane S3, *G. parasuis* NO635; lane S4, Pasteurella multocida CGKV-VM02; lane S5, Streptococcus suis HB27; lane S6, Mycoplasma hyopneumoniae HN17; lane S7, enterotoxigenic Escherichia coli DDH135; lane S8, Salmonella Typhimurium CVCC542; lane S9, Staphylococcus aureus ATCC 25923; lane H_2_O, negative control. (b) Comparison of between previously reported single-plex PCR and our multiplex PCR. Sample types are as follows: 1, samples carrying S. suis; 2, carrying A. pleuropneumoniae and S. suis; 3, carrying *G. parasuis* and S. suis; 4, carrying A. pleuropneumoniae, *G. parasuis*, and S. suis; 5, carrying *G. parasuis*; 6, carrying A. pleuropneumoniae and *G. parasuis*.

Specific amplification bands were obtained and verified by DNA sequencing after single-plex PCR amplification. The single-plex PCR results showed that the target genes were successfully amplified by using specific primers, indicating that these primers were qualified for multiplex PCR assay. As expected, our designed primers were also found to be specific using multiplex PCR, and non-target strains displayed no evidence of fragment amplification (Fig. 2a and Fig. S1 in the supplemental material). These non-target strains used for PCR amplification were isolated from pig farms and identified by 16S rRNA sequencing.

### Comparison of detection results between single-plex PCR and multiplex PCR.

A total of 78 clinical samples randomly selected were subjected to PCR assay, and the detection results were compared between our multiplex PCR method and previously reported single-plex PCR (27–31). The results showed that the consistency between multiplex PCR and single-plex PCR reached 94.87% (74/78) (Fig. 2b). Of these 78 samples, 13 carried S. suis; 10 carried A. pleuropneumoniae and S. suis; 19 carried *G. parasuis* and S. suis; 25 carried A. pleuropneumoniae, *G. parasuis*, and S. suis; 3 carried *G. parasuis*, 7 carried A. pleuropneumoniae and *G. parasuis*; and 1 carried undetected (UD) target bacteria.

### Epidemic situation and characteristics of the PRBPs in Guangxi Province.

**FIG 3:**
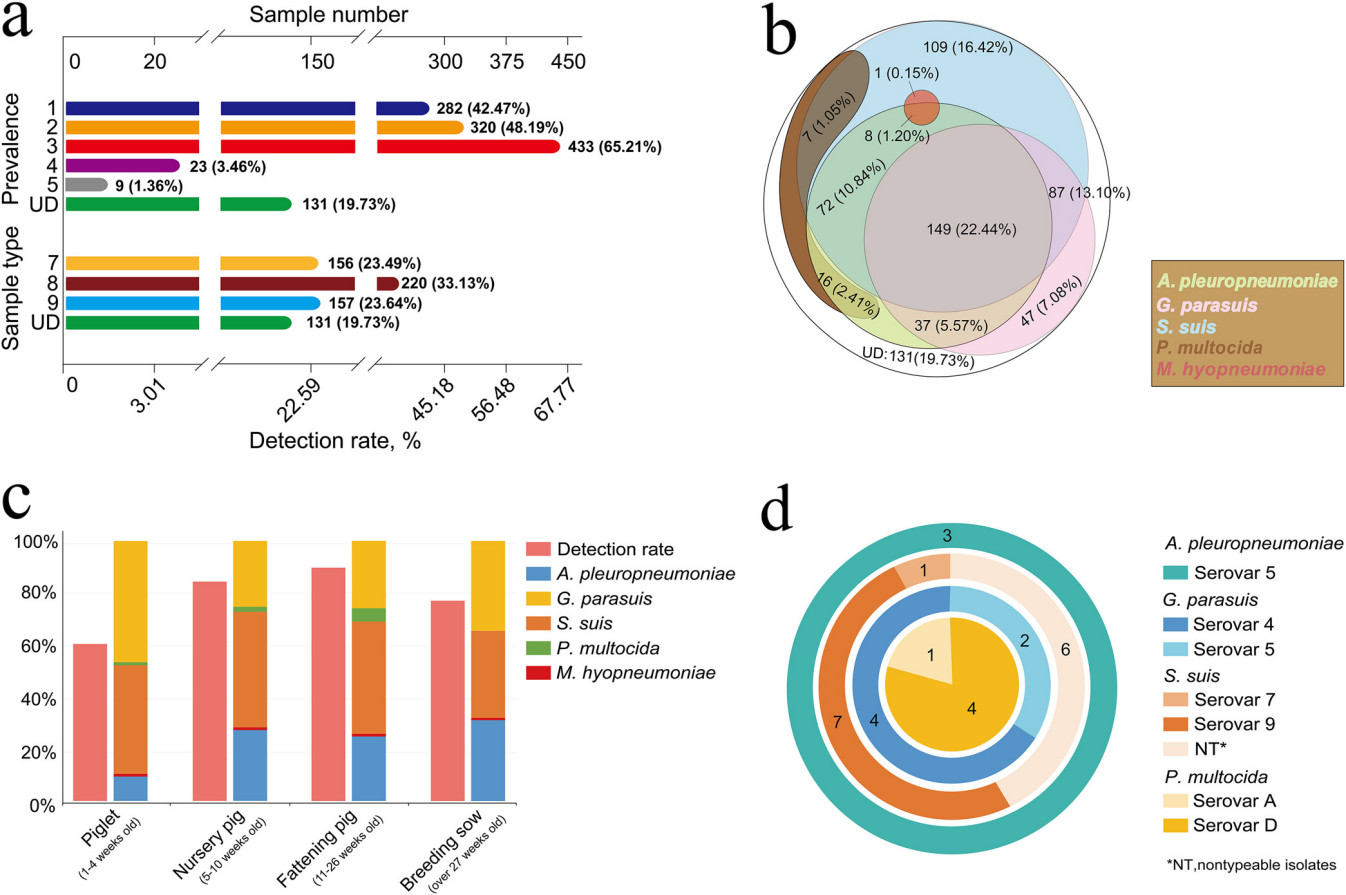
Sample diagnostic results. (a) Statistics of diagnostic results of samples. Prevalences: 1, samples carrying A. pleuropneumoniae; 2, samples carrying *G. parasuis*; 3, samples carrying S. suis; 4, samples carrying P. multocida; 5, samples carrying M. hyopneumoniae. Sample types: 7, carrying one type of bacteria; 8, carrying two types of bacteria; 9, carrying three types of bacteria; UD (undetected), samples carrying no target bacteria. (b) Numbers and types of samples carrying targeted bacteria. (c) Detection rates and proportions of pathogenic bacteria in pigs at different stages (age). (d) Numbers and serovars of isolates from positive samples.

To examine the epidemiological characteristics of PRBPs in Guangxi Province, we tested a total of 664 nasal swabs using multiplex PCR. The results showed that 433 (accounting for 65.21%) of the 664 nasal swab samples carried S. suis, and similar numbers of samples carried *G. parasuis* (320, 48.19%) and A. pleuropneumoniae (282, 42.47%). However, only a few samples were found to carry P. multocida and M. hyopneumoniae: 23 (3.46%) for P. multocida and 9 (1.36%) for M. hyopneumoniae. In addition, 131 (19.73%) of the 664 swabs were found to carry no target bacteria (Table 1 and Fig. 3a), indicating that these samples might have been from pigs infected with respiratory viral pathogens or uninfected pigs, rather than from pigs infected with bacterial pathogens. A total of 220 (33.13%) of the 664 nasal swabs carried two types of target bacteria, 157 (23.64%) carried three types of bacteria, and 156 (23.49%) carried only one type of bacteria (Table 2). The 10 types of nasal swabs carrying different bacteria and their numbers are shown in Table 3 and Fig. 3a and b. The proportion of nasal swabs carrying S. suis, *G. parasuis*, and A. pleuropneumoniae was the highest (149, 22.44%), which indicated that these frequently co-infected swine herds in Guangxi Province. Multiple types and a large number of nasal swab samples carried S. suis: 109 (16.42%) of nasal swabs carried only S. suis, 87 (13.10%) carried S. suis and *G. parasuis*, and 72 (10.84%) carried S. suis and A. pleuropneumoniae. In contrast, relatively few samples carried P. multocida and M. hyopneumoniae. Our results suggested that S. suis was the most prevalent respiratory bacterium among pigs with respiratory symptoms in Guangxi province and played an important role in co-infection.

**TABLE 1:** Summary of sample detection results and information on isolates^*a*^

Species	No. of samples and detection rate (%)	Isolate serotype (*n*)
A. pleuropneumoniae	282 (42.47)	5 (3)
*G. parasuis*	320 (48.19)	4 (4)
		5 (2)
S. suis	433 (65.21)	7 (1)
		9 (7)
		NT (6)
P. multocida	23 (3.46)	A (1)
		D (4)
M. hyopneumoniae	9 (1.36)	
UD	131 (19.73)	

a UD (undetected), samples carrying no target bacteria; NT, non-typeable isolates.

**TABLE 2:** Type and number of samples carrying bacteria^*a*^

Sample type	*N* (%)
Single	156 (23.49)
Dual	220 (33.13)
Triple	157 (23.64)
UD	131 (19.73)
Total	664 (100)

a Single, samples carrying one type of target bacteria; Dual, samples carrying two types; triple, samples carrying three types; UD (undetected), samples carrying no target bacteria.

**TABLE 3:** Type and number of samples from pigs at different growth stages^*a*^

Sample type	Source (*n*)				
	Piglet	Nursery pig	Fattening pig	Breeding sow	Total (%)
A + G + S	6	40	37	66	149 (22.44)
A + S + M	1	2	3	2	8 (1.20)
A + G		13	6	18	37 (5.57)
A + P	1	3	12		16 (2.41)
A + S	1	46	15	10	72 (10.84)
G + S	20	34	23	10	87 (13.10)
M + S		1			1 (0.15)
P + S		4	3		7 (1.05)
G	16	8	11	12	47 (7.08)
S	10	37	49	13	109 (16.42)
UD	36	36	19	40	131 (19.73)
Total	91	224	178	171	664 (100)

a A, A. pleuropneumoniae; G, *G. parasuis*; S, *S.suis*; P, P. multocida; M, M. hyopneumoniae; UD (undetected), samples carrying no target bacteria.

The detection rates for pathogenic bacteria in piglets (1 to 4 weeks old), nursery pigs (5 to 10 weeks old), fattening pigs (11 to 26 weeks old), and breeding sows (over 27 weeks old), respectively, were 60.44%, 83.93%, 89.33%, and 76.61%. Our results indicated that the detection rate and proportion of pathogenic bacteria varied with different pig growth stages (Fig. 3c).

### Isolation of PRBPs.

The 28 isolates of A. pleuropneumoniae, *G. parasuis*, S. suis, and P. multocida were obtained from 533 positive samples, but no isolates of M. hyopneumoniae were available. These isolates were serotyped by specific serotype primers: only 4 representative virulent serovars (serovars 1, 2, 7, and 9) of S. suis were identified due to the presence of up to 29 serovars. The results showed that 14 strains of S. suis were isolated, including 1 of serovar 7, 7 of serovar 9, and 6 unserotyped isolates. The numbers of serotyped isolates for *G. parasuis* and P. multocida were similar: 6 (4 of serovar 4, 2 of serovar 5) for *G. parasuis* and 5 (1 of serovar A, 4 of serovar D) for P. multocida. The 3 isolates of A. pleuropneumoniae were identified as serovar 5 (Table 1 and Fig. 3d).

### Antibiotic susceptibility profile of isolates.

**FIG 4:**
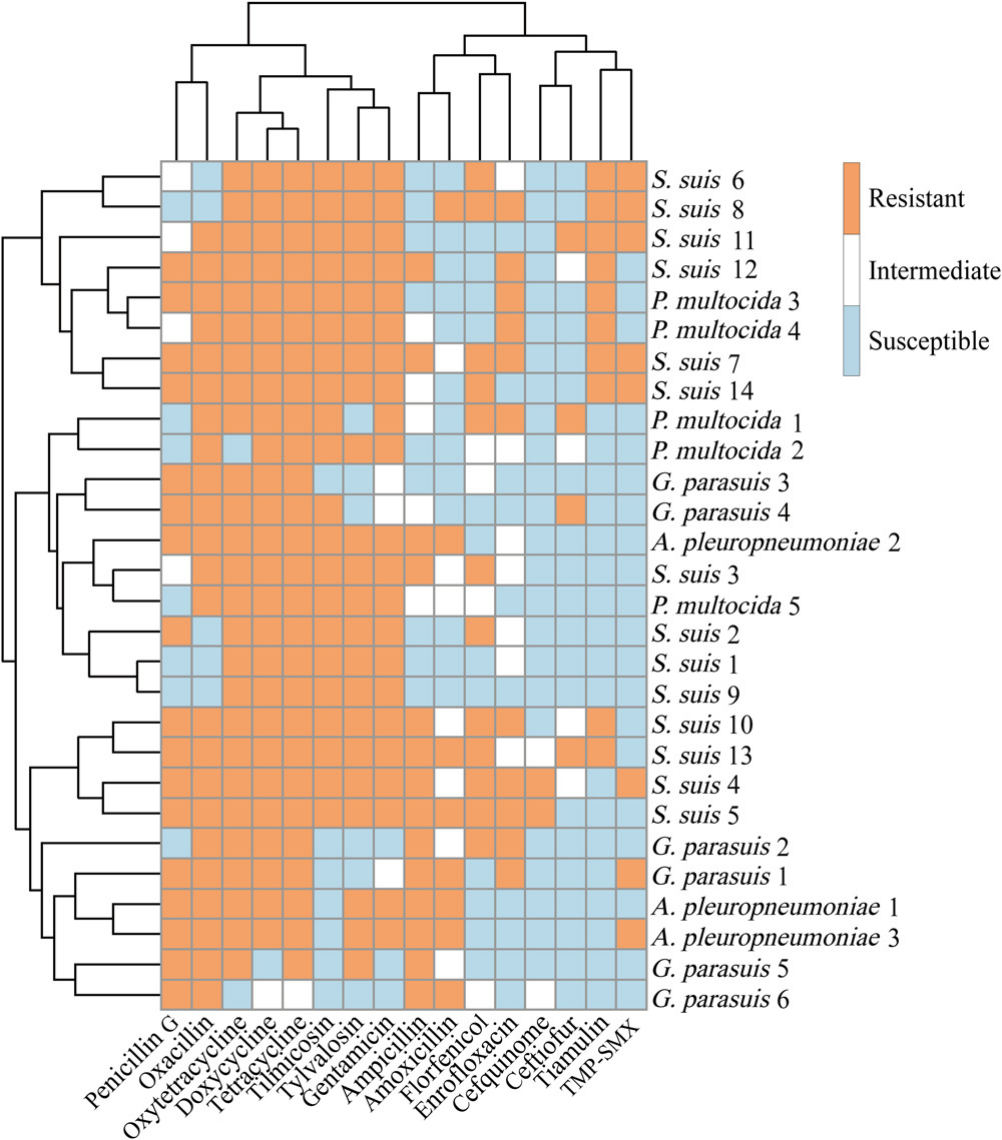
Susceptibility of isolates to 16 antibiotics.

Our antibiotic susceptibility testing (AST) results revealed that 24 (85.71%) of the 28 isolates of A. pleuropneumoniae, *G. parasuis*, S. suis, and P. multocida were sensitive to the antibiotic cefquinome. More than 18 (64.29%) of the 28 isolates were sensitive to ceftiofur, TMP-SMX, and tiamulin antibiotics. In contrast, more than 21 (75%) of the 28 isolates were resistant to oxacillin, oxytetracycline, doxycycline, tetracycline, tilmicosin, tylvalosin, and gentamicin. Antibiotic susceptibility details and MIC values are shown in Fig. 4 and Tables S1 and S2.

## DISCUSSION

PRDC has become a bottleneck for large-scale pig farms. Due to the similar clinical signs of different pathogen infections, the identification of pathogens causing PRDC poses a challenge to most pig farms, making it impossible to apply targeted preventive measures against unknown pathogens. In this study, we developed a multiplex PCR method for detecting common PRBPs, mainly including A. pleuropneumoniae, *G. parasuis*, S. suis, P. multocida, and M. hyopneumoniae, and conducted the first systematic investigation of the prevalence of these PRBPs in Guangxi province. A total of 664 samples from pigs with respiratory symptoms were analyzed in this study. The results showed that S. suis (65.21%) was the most dominant bacterium causing PRDC in Guangxi Province, followed by *G. parasuis* (48.19%) and A. pleuropneumoniae (42.47%). These 3 types of bacteria tended to co-infect swine herds. In addition, 85.71% of the isolates were sensitive to the antibiotic cefquinome, followed by ceftiofur (71.43% of the isolates), TMP-SMX (71.43%), and tiamulin (64.29%). Notably, although our results were not intended for clinical medication guidance, they were of great significance for revealing the epidemiological characteristics of PRBPs in Guangxi Province and for subsequent research and development of clinical treatment drugs.

Our results revealed that S. suis, *G. parasuis*, and A. pleuropneumoniae were the top 3 dominant bacteria causing PRDC in Guangxi Province, and they frequently co-infected swine herds. Although the detection rate of A. pleuropneumoniae was not as high as that of S. suis and *G. parasuis*, considering the history of A. pleuropneumoniae outbreaks in some pig farms, we initially speculated that A. pleuropneumoniae infected the swine herds first, reducing overall herd resistance, and eventually potentially caused the secondary infection of S. suis and *G. parasuis*. Our speculation was supported by previous reports in which mixed infections with low-pathogenic bacteria had potential to cause severe respiratory disease (32–34). These findings imply that pig herds are more susceptible to co-infection once they have been attacked by pathogens. Considering this, we suggest that A. pleuropneumoniae removal should be given priority in local measures to prevent and control respiratory disease in pigs. In contrast, P. multocida and M. hyopneumoniae were the least frequently detected bacteria in this study. This may be because there were relatively small numbers of these two pathogens in the environment or because the herds had been vaccinated against these two pathogens. Nasal swabs may not be effective at collecting M. hyopneumoniae because M. hyopneumoniae lives deep in the lungs and is only intermittently removed from the nose (35). Furthermore, more research is needed to further construct phylogenetic trees for A. pleuropneumoniae, *G. parasuis*, S. suis, and P. multocida for evolutionary analysis. However, M. hyopneumoniae was not detected in isolates, possibly due to a low detection rate, highly demanding culture conditions, the presence of numerous non-target bacteria on the culture plates, and long-term transportation. In addition, these samples (where no target bacteria were detected) may have been from swine herds infected with viruses such as PRRSV, PCV2, and SIV, which remains to be investigated in future studies.

Our results were generally consistent with a report by MacInnes et al. (36) that the detection rates of S. suis, *G. parasuis*, and A. pleuropneumoniae reached 78%, 96%, and 98%, respectively, in 50 Canadian swine herds. The similar bacterial epidemiological characteristics between Ontario, Canada and Guangxi, China may suggest a common mechanism of S. suis, *G. parasuis*, and A. pleuropneumoniae co-infection. This co-infection mechanism remains poorly understood and might be the focus of our future studies. Interestingly, Zhu et al. (37) reported that detection rates of S. suis, *G. parasuis*, and A. pleuropneumoniae were 71%, 55.6%, and 0% in samples from healthy pigs in eastern China. The large difference in the A. pleuropneumoniae detection rate between their study and ours (0% versus 42.47%) may suggest that A. pleuropneumoniae plays an important role in the development of respiratory symptoms in pigs. Based on these findings, we speculated that healthy pigs carry small amounts of S. suis and *G. parasuis* asymptomatically but might develop respiratory symptoms due to A. pleuropneumoniae exposure-weakened resistance. The detection rates of S. suis, *G. parasuis*, and A. pleuropneumoniae in 14 provinces in China as reported by Zhang et al. (38) was generally consistent with our results. Unlike in their study, we focused on Guangxi Province and only collected samples from pigs with respiratory symptoms, which made our findings more relevant to pathogens. To our knowledge, this is the first report on the prevalence of PRBPs in Guangxi Province, China, which provides important reference for subsequent PRDC prevention and control.

The lowest detection rate (60.44%) of pathogenic bacteria was found in piglets (1 to 4 weeks old). The reason for this low detection rate in piglets might be attributed to our sampling method. During sampling, we sampled only the surviving piglets protected by antibodies from breast milk and excluded those which might have died from pathogen infection.

In this study, we investigated the sensitivity of clinical strains to the antibiotics commonly used in local pig farms in Guangxi Province, China. Our results demonstrated that most of the isolates were sensitive to the antibiotics cefquinome, ceftiofur, TMP-SMX, and tiamulin. In contrast, most of the isolates were resistant to oxacillin, oxytetracycline, doxycycline, tetracycline, tilmicosin, tylvalosin, and gentamicin. Recent studies have found that over 90% of S. suis isolates (from 2005 to 2014) were resistant to tetracyclines, which is consistent with our study (39–41). However, European isolates of S. suis (from 1967 to 1981) were reported to be non-resistant to tetracyclines (42–44). These inconsistent reports might be because long-time application of tetracyclines against PRDC has resulted in the generation of drug resistance. This evolution of strains from antibiotic sensitivity to resistance has resulted from the abuse of antibiotics (45, 46). Therefore, the management of antibiotic application should be strengthened in the future. One antibiotic sensitivity test in Jiangsu Province, China indicated that S. suis was susceptible to chloramphenicol (17). However, chloramphenicol was not selected in this study because it had been clinically banned in China. Additionally, we only isolated 28 strains of PRBPs from the 533 positive samples, which may be due to the PRBPs’ highly demanding culture conditions, the existence of numerous non-target bacteria on the culture plates, and bacterial death from long-term transportation. The MIC values for these isolates may only represent the antibiotic susceptibility profiles of some local strains, and more isolates are needed to determine the antibiotic susceptibility profiles of local strains in the future.

Considering the interference between the primers based on the *apxIV* gene of A. pleuropneumoniae and those based on other pathogens, we chose only the conserved region in the 16S rRNA gene of A. pleuropneumoniae for primer design. In a laboratory environment, the single template was amplified to obtain a specific band. However, during clinical sample detection, non-target bands appeared occasionally and irregularly depending on the complexity of the templates, which may be attributed to the mismatches between different primers. This amplification of non-target genes is very common in multiplex PCR. In this study, the non-target bands we found were far from the target bands, and thus they did not affect the determination of positive samples. The identification of multiple serotypes and pathogens using one PCR deserves further efforts, which will contribute to improving the efficiency of prevention and treatment of swine diseases.

In conclusion, this study developed a multiplex PCR method targeting PRBPs, including A. pleuropneumoniae, *G. parasuis*, S. suis, P. multocida, and M. pneumoniae. We found that the dominant bacterial pathogens causing PRDC in Guangxi were S. suis, *G. parasuis*, and A. pleuropneumoniae, which frequently co-infected swine herds. AST results demonstrated that 85.71% of the isolates were sensitive to cefquinome, followed by ceftiofur (71.43% of the isolates), TMP-SMX (71.43%), and tiamulin (64.29%). More than 75% of the isolates were resistant to oxacillin, oxytetracycline, doxycycline, tetracycline, tilmicosin, tylvalosin, and gentamicin. Our findings provide new information on the prevalence of PRBPs in Guangxi and offer a reference for the development of local clinical therapeutic drugs. Our work contributes to understanding the distribution of PRBPs in China and complements the global PRBP prevalence database. Our research has important implications for public health and the development of the pig industry.

## MATERIALS AND METHODS

### Bacterial strains.

All strains employed in this study, including A. pleuropneumoniae L20, *G. parasuis* NO635, S. suis HB27, P. multocida CGKV-VM02, and M. hyopneumoniae HN17 were provided by the State Key Laboratory of Agricultural Microbiology, Huazhong Agricultural University.

### DNA extraction.

Genomic DNA was extracted from bacteria preserved in the laboratory utilizing a TIANamp Bacteria DNA kit (Tiangen, China). The concentrations of genomic DNA extracts were measured using an ultra-micro spectrophotometer (Thermo Fisher Scientific, USA) and were diluted to 50 ng/μL. The 1-μL DNA mixture contained 10 ng each of A. pleuropneumoniae L20, *G. parasuis* NO635, S. suis HB27, P. multocida CGKV-VM02, and M. hyopneumoniae HN17 genomic DNA. The genomic DNA extracts and this DNA mixture were utilized for subsequent PCR amplification.

### Single-plex PCR.

Specific primers located in a conserved gene for each strain were designed. Primer sequences, annealing temperatures, PCR product sizes, and information on A. pleuropneumoniae, *G. parasuis*, S. suis, P. multocida, and M. hyopneumoniae are provided in Table 4. PCR was performed with a Bio-Rad S1000 thermal cycler (Bio-Rad, USA) in a 20-μL reaction system containing 10 μL of 2× *Taq* PCR Master Mix (Vazyme, China), 0.5 μL of forward and reverse primers (10 μM), 8 μL sterile water, and 1 μL bacterial genomic DNA extract. The commercial reagent 2× *Taq* PCR Master Mix contained *Taq* DNA polymerase, deoxynucleoside triphosphate, MgCl_2_, PCR buffer, PCR stabilizers, gel loading reagent, and bromophenol blue dye. PCR was performed as follows: pre-denaturation at 94°C for 5 min, 30 cycles of denaturation at 94°C for 30 s, annealing at 5 optimal temperatures (Table 4) for 30 s, and extension at 72°C for 30 s, followed by a final extension at 72°C for 10 min. The PCR products were separated through 1.0% agarose gel. Products of single-plex PCR amplification were sequenced to ensure the accuracy of amplification. After repeated verification of single-plex PCR specificity, a multiplex PCR method was established.

**TABLE 4:** Primers used for multiplex PCR

Species and strain	Strain	Target gene	Direction	Sequence (5′→3′)	Product size (bp)	Temp (°C)	Primer location (position)
A. pleuropneumoniae	L20	*16S rRNA*	Forward	ATGGCTCAGATTGAACGC	562	54.8	19−580
			Reverse	GCCCTTTACGCCCAGTTA			
*G. parasuis*	NO635	*16S rRNA*	Forward	GACGGGAAACTGTCGCTAA	979	56.4	149−1,127
			Reverse	TCGCTGGCAACAAAGGAT			
S. suis	HB27	*GDH*	Forward	CACATCGGACCTTCACTT	220	50.3	274−493
			Reverse	AGGATTTACCGTTTGCTG			
P. multocida	CGKV-VM02	*KMT−1*	Forward	ATCCGCTATTTACCCAGTG	123	52.6	1−123
			Reverse	GACTACCGACAAGCCCAC			
M. hyopneumoniae	HN17	*P36*	Forward	TTCCGATTAGTGTCTCCC	294	54.0	170−463
			Reverse	ATTGAAGCCTTGCTGTAT			

### Multiplex PCR.

Multiplex PCR parameters were optimized at different annealing temperatures by a gradient PCR assay. After multiple rounds of experiments, the optimal PCR amplification conditions were determined. Multiplex PCR was conducted in a 30-μL reaction system containing 15 μL of 2× *Taq* PCR Master Mix, 3 μL sterile water, 1 μL of forward and reverse primers (10 μM), and 2 μL of DNA mixture. Multiplex PCR was performed as follows: pre-denaturation at 95°C for 10 min, 30 cycles of denaturation at 95°C for 30 s, annealing at 53.8°C for 30 s, and an extension at 72°C for 30 s, followed by a final extension at 72°C for 10 min. Multiplex PCR experiments were repeated twice, and PCR products were separated by gel electrophoresis on a 1.0% agarose gel.

### Evaluation on specificity and accuracy of multiplex PCR assay.

The specificity of the multiplex PCR assay was evaluated through amplification of the genomic DNA extracts described above. To verify the accuracy of the method we established in this experiment, we compared our multiplex PCR method with PCR methods reported in the literature (Table S3) (27–31). The 78 clinical samples were randomly selected as the templates and subjected to both multiplex PCR and the previously described single-plex PCR assays.

### Clinical samples.

To investigate the epidemiological characteristics of PRBPs in Guangxi Province, a total of 664 nasal swab samples were collected from pigs with respiratory symptoms in 42 farms in Guangxi Province by our laboratory members with assistance from the veterinary diagnostic laboratory of Yangxiang Co., Ltd., and assayed by the multiplex PCR method described. Specifically, nasal swabs were inserted into the noses of pigs to stimulate them to sneeze. Subsequently, these nasal swab samples were preserved in centrifuge tubes filled with sterile saline.

### Isolation of strains from nasal swabs.

Positive nasal swabs were smeared into culture plates with tryptic soy agar solid medium (TSA, Difco Laboratories, Detroit, MI) containing 0.05% NAD (BioFroxx) and 5% fetal bovine serum (Sigma-Aldrich) and cultured at 37°C for 12 h. Afterwards, single colonies with different appearances were selected and cultured in tryptic soy broth medium (TSB, Difco Laboratories, Detroit, MI) containing 0.05% NAD and 5% fetal bovine serum. The cells were grown at 37°C until the optical density at 600 nm (OD_600_) reached 0.8. Subsequently, multiplex PCR, Gram-staining, and 16S rRNA sequencing were performed to identify isolate species. Next, the serotypes of these isolates were further identified with specific serotype primers (Tables S4 to S7). We only identified representative virulent serotypes of S. suis due to the presence of up to 29 serotypes (47).

### Antibiotic susceptibility test.

The 28 isolates of A. pleuropneumoniae, *G. parasuis*, S. suis, and P. multocida were selected for antibiotic susceptibility testing. The MIC assays were performed following Clinical and Laboratory Standards Institute (CLSI) guidelines to investigate the antibiotic susceptibility of isolates. We tested the resistance of isolates to 16 antibiotics. Briefly, the 28 isolates were cultured in media containing different concentrations of antibiotics at 37°C for 24 h. The lowest concentration of antibiotics at which no visible bacterial growth was observed was regarded as the MIC. This study used the following 16 antibiotics: cefquinome, ceftiofur, TMP-SMX, tiamulin, oxacillin, oxytetracycline, doxycycline, tetracycline, tilmicosin, tylvalosin, gentamicin, penicillin G, ampicillin, amoxicillin, florfenicol, and enrofloxacin. TMP-SMX was used in combination at a ratio of 1:19 and diluted according to the concentration of TMP (128 μg/mL to 0.06125 μg/mL) in the MIC assays. MIC values were recorded. The susceptibilities of all isolates to these 16 antibiotics were classified into 3 categories (resistant, intermediate, and susceptible isolates) according to the MIC breakpoints provided by published literature and the CLSI.

### Analytical methods.

Microsoft Office Excel 2019 was used for data processing and analysis. Statistical charts and tables were used to describe the overall trend of PRBP prevalence in Guangxi Province, the number and proportion of pathogens detected, the type and composition ratio of pathogens, the serotype and number of isolates, and the MIC values of 16 antibiotics against all isolates.
