# Supplementary material for: Novel Multiplex PCR Assay and Its Application in Detecting Prevalence and Antibiotic Susceptibility of Porcine Respiratory Bacterial Pathogens in Guangxi, China
Source: Microbiol Spectr. 2023 Mar 14;11(2):e03971-22. doi: 10.1128/spectrum.03971-22 (PMC10100844; doi:10.1128/spectrum.03971-22)
Supplement: Supplemental file 1 — Supplemental material. Download spectrum.03971-22-s0001.pdf, PDF file, 0.3 MB [file spectrum.03971-22-s0001.pdf]

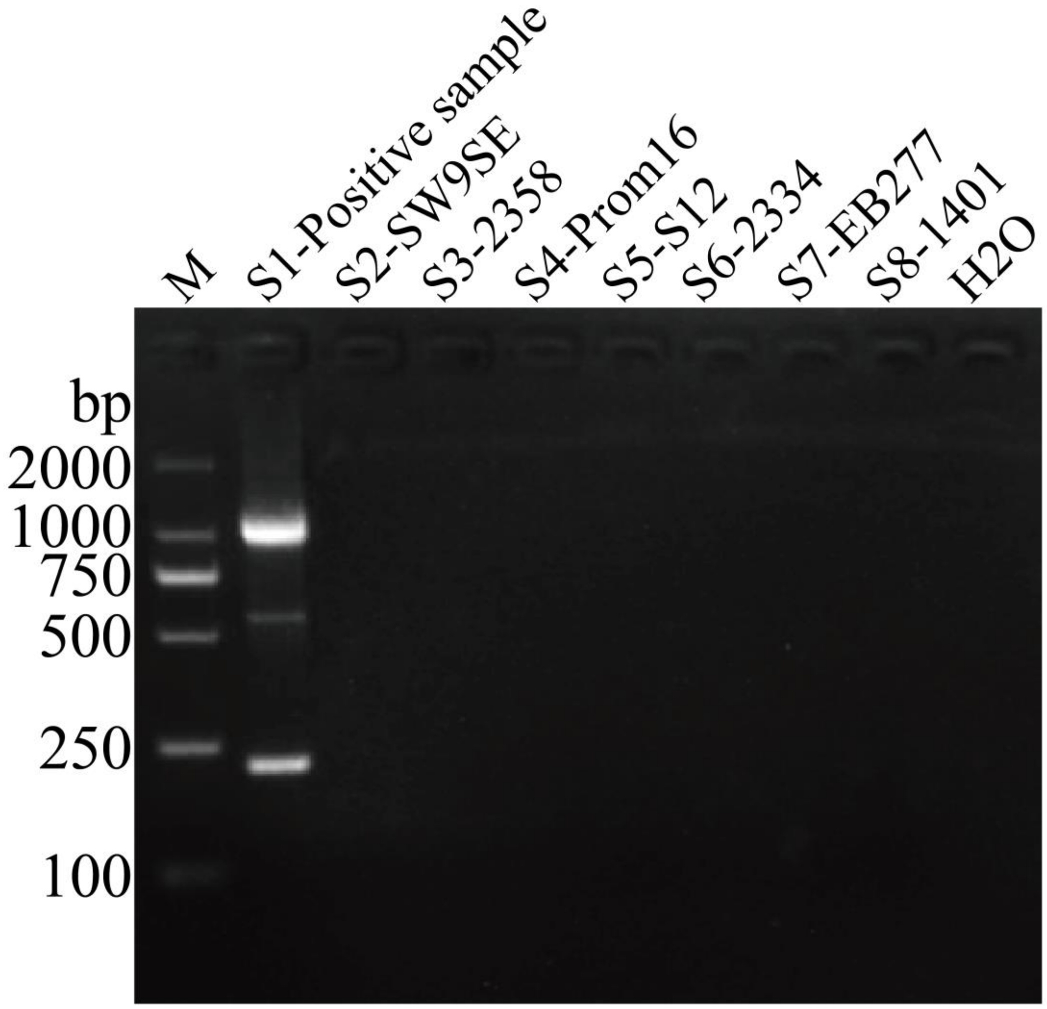

FIG S1 Specificity evaluation of multiplex PCR. lane M, DL2000 DNA molecular weight marker; Lane S1, samples carrying *A. pleuropneumoniae*, *G. parasuis*, and *S. suis*; lane S2, *Bacillus cereus* SW9SE; lane S3, *Enterococcus faecalis* 2358; lane S4, *Proteus mirabilis* Prom16; lane S5, *Lactococcus lactis* S12; lane S6, *Leuconostoc lactis* 2334; lane S7, *Pseudomonas fulva* EB277; lane S8, *Staphylococcus chromogenes* 1401; lane H_2O, negative control.

Supplemental Table S1 Statistics on the susceptibility of isolates to 16 antibiotics

	Antibiotics	Isolate number, proportion		
		**Resistant**	**Intermediate**	**Susceptible**
1	Tiamulin	10, 35.71%	0	18, 64.29%
2	Tylvalosin	22, 78.57%	0	6, 21.43%
3	Penicillin G	17, 60.71%	4, 14.29%	7, 25.00%
4	Doxycycline	26, 92.86%	1, 3.57%	1, 3.57%
5	Tilmicosin	21, 75.00%	0	7, 25.00%
6	Gentamicin	22, 78.57%	3, 10.71%	3, 10.71%
7	Ampicillin	14, 50.00%	5, 17.86%	9, 32.14%
8	Oxacillin	23, 82.14%	0	5, 17.86%
9	Florfenicol	12, 42.86%	4, 14.29%	12, 42.86%
10	Enrofloxacin	11, 39.26%	7, 25.00%	10, 35.71%
11	Amoxicillin	8, 28.57%	7, 25.00%	13, 46.43%
12	Cefquinome	2, 7.14%	2, 7.14%	24, 85.71%
13	Ceftiofur	4, 14.29%	4, 14.29%	20, 71.43%
14	Oxytetracycline	26, 92.86%	0	2, 7.14%
15	Tetracycline	27, 96.43%	1, 3.57%	0
16	TMP-SMX	8, 28.57%	0	20, 71.43%

Supplemental Table S2 The MIC values of isolates to 16 antibiotics

	Isolates*	Antibiotics (MIC, µg/mL)								
		**1. Tiamulin**	**2. Tylvalosin**	**3. Penicillin G**	**4. Doxycycline**	**5. Tilmicosin**	**6. Gentamicin**	**7. Ampicillin**	**8. Oxacillin**	**9. Florfenicol**
1	A1	16	128	1	8	16	16	4	4	0.25
2	A2	8	64	4	4	32	8	4	4	0.5
3	A3	4	32	8	2	8	16	4	2	0.25
4	G1	4	16	4	4	2	4	2	4	1
5	G2	4	16	0.25	8	4	2	4	4	8
6	G3	4	8	1	4	4	4	0.25	2	4
7	G4	4	4	8	2	32	4	0.5	4	0.25
8	G5	8	32	4	0.25	16	2	4	8	2
9	G6	8	8	8	1	8	2	2	4	4
10	S1	0.25	128	0.06125	64	256	64	0.06125	0.06125	1
11	S2	0.25	64	1	64	256	32	0.06125	0.06125	32
12	S3	1	64	0.5	32	256	16	8	4	8
13	S4	1	128	8	32	256	32	8	4	16
14	S5	2	256	16	32	256	128	8	4	16
15	S6	128	128	0.5	16	256	32	0.25	0.25	16
16	S7	256	256	8	64	256	128	8	8	32
17	S8	128	256	0.06125	64	256	64	0.125	0.25	8
18	S9	0.5	256	0.06125	8	256	8	0.06125	0.125	1
19	S10	128	256	16	32	256	16	8	8	32
20	S11	128	256	0.5	16	256	16	0.06125	2	0.5
21	S12	256	64	8	32	256	64	4	4	2
22	S13	128	128	16	32	256	64	16	8	8
23	S14	256	256	16	16	256	64	0.5	2	8
24	P1	8	16	0.25	8	256	64	1	2	8
25	P2	4	32	0.06125	4	256	64	0.25	2	4
26	P3	256	256	1	32	256	256	0.25	4	1
27	P4	256	256	0.5	32	256	256	0.5	4	1
28	P5	4	256	0.25	16	256	128	0.5	2	4

*A, *A. pleuropneumoniae*; G, *G. parasuis*; S, *S.suis*; P, *P. multocida*.

	Isolates*	Antibiotics (MIC, μg/mL)						
		10. Enrofloxacin	11. Amoxicillin	12. Cefquinome	13. Ceftiofur	14. Oxytetracycline	15. Tetracycline	16. TMP-SMX
1	A1	0.06125	4	0.06125	0.06125	8	8	0.5
2	A2	0.5	4	0.06125	0.125	4	4	0.25
3	A3	0.125	2	0.125	0.125	8	16	4
4	G1	2	2	1	0.5	16	16	4
5	G2	2	1	0.125	0.5	2	4	0.125
6	G3	0.06125	0.5	0.125	2	4	8	0.125
7	G4	0.06125	0.25	0.25	8	32	2	0.25
8	G5	0.125	1	0.25	1	2	2	2
9	G6	0.06125	2	4	2	0.5	1	1
10	S1	1	0.06125	0.06125	0.06125	8	64	1
11	S2	1	0.06125	1	1	8	64	2
12	S3	1	1	0.5	1	8	64	0.125
13	S4	2	1	8	4	16	16	8
14	S5	2	2	16	8	32	16	2
15	S6	1	0.06125	0.5	1	8	8	4
16	S7	2	1	1	2	32	64	4
17	S8	2	2	1	0.25	4	32	8
18	S9	0.125	0.06125	0.06125	0.25	4	8	0.5
19	S10	4	1	0.25	4	128	32	0.5
20	S11	0.125	0.06125	0.125	16	32	16	4
21	S12	2	0.06125	0.25	4	4	16	1
22	S13	1	4	4	8	4	16	0.25
23	S14	0.5	0.06125	0.125	0.25	32	16	4
24	P1	16	0.5	2	8	8	16	0.125
25	P2	0.5	0.5	0.5	4	0.25	16	0.125
26	P3	32	0.06125	0.25	1	16	32	0.5
27	P4	32	0.06125	0.125	0.25	16	32	2
28	P5	0.25	1	0.06125	0.25	4	16	0.06125

*A, *A. pleuropneumoniae*; G, *G. parasuis*; S, *S.suis*; P, *P. multocida*.

Supplemental Table S3 Primers reported in the literature for the detection of porcine respiratory bacterial pathogens

Species	Direction	Targeted gene	Sequence (5′to 3′)	Product size，bp	Temp，°C	Reference
A. pleuropneumoniae	Forward<br>Reverse	*apxIV*	TTATCCGAACTTTGGTTTAGCC<br>CATATTTGATAAAACCATCCGTC	418	57	Zhou et al.（1）
G. parasuis	Forward<br>Reverse	*HPS_219690793*	ACAACCTGCAAGTACTTATCGGGAT<br>TAGCCTCCTGTCTGATATTCCCACG	275	58	Howell et al.（2）
S. suis	Forward<br>Reverse	*gdh*	TTCTGCAGCGTATTCTGTCAAACG<br>TGTTCCATGGACAGATAAAGATGG	695	58	Kerdsin et al.（3）
P. multocida	Forward<br>Reverse	*KMT1*	ATCCGCTATTTACCCAGTGG<br>GCTGTAAACGAACTCGCCAC	457	56	Peng et al.（4）
M. hyopneumoniae	Forward<br>Reverse	*16S rRNA*	CTGGCTCAGATAAACGCTAG<br>GCTGTGTCGCTCCATCAAG	347	60	Qiu et al.（5）

Supplemental Table S4 Primers for serotyping of *A. pleuropneumoniae*

Serotype	Direction	Targeted gene	Sequence (5′to 3′)	Product size，bp	Temp，°C	Reference
1	Forward	*cps1B*	CTGGAGTAATTACGGCGACTATTCC	959	58	Bossé et al.（6）
	Reverse		AGGAGAAGCTAGTAGTACTTGCATTTTC			
2	Forward	*cps2E*	GAGTGTGATGATGATGCTCTGGTTC	247	58	Bossé et al.（6）
	Reverse		TACCAATAACTGTTGCAACTAACGC			
3	Forward	*cps3F*	TTGTAGAGCCCGCCAGATTTACG	500	58	Bossé et al.（7）
	Reverse		CATTCGCACCAGCAATCACC			
4	Forward	*cps4B*	CAGCATGGGTTTGGTCCTGTTG	204	58	Bossé et al.（7）
	Reverse		GGCTTTCTCCGTGTATGAATAAAGTG			
5	Forward	*cps5B*	AGCCACAAGACCCGAATGGTATAATG	825	58	Bossé et al.（6）
	Reverse		CCATCAAATGCAGCTTCAAGGAGC			
6	Forward	*cps6F*	TGACTGGCTTCGTGAAAATGAG	718	58	Bossé et al.（7）
	Reverse		GTCTGAAGTTTTATTCGCAGCTCC			
7	Forward	*cps7E*	TCTAGGTATTACTGGTGTTCCTGATG	601	58	Bossé et al.（6）
	Reverse		CGTCCAACACGAGCAACTACG			
8	Forward	*cps8F*	ACATCCAAGCCGTTCTCCAG	1126	58	Bossé et al.（7）
	Reverse		CATCCATGAGCCAATGAGGG			
9	Forward	*cps9E*	GTAGGACGTGGTAACATTGAGGC	2105	58	Bossé et al.（7）
	Reverse		ACGGGTGCAATTTCTAAAGCTG			
10	Forward	*cps10A*	GGTGGTGATGGAACAAGGTTATGG	183	58	Bossé et al.（6）
	Reverse		CTGTAATTGATGCGAAATAGTAGATTGGTGC			
12	Forward	*cps12A*	TAAAGGTATTATAACGCCGGCTCT	347	58	Bossé et al.（6）
	Reverse		CTCCCATCTGTTGTCTAAGTAGTAG			
13	Forward	*cps13E*	GTTGTGTATCGAGGTTGGCATTTC	665	58	Bossé et al.（7）
	Reverse		ATGTAAAGGATCTAAGCCGTGTG			
14	Forward	*cps14G*	TGCATTACGCTTATATTCTGAATGG	1911	58	Bossé et al.（7）
	Reverse		TTGTCGATCGAGAGGGAGTAACG			
15	Forward	*cps15B*	GCAACTTGGAGAACATGGTTAAATCAAG	1595	58	Bossé et al.（7）
	Reverse		CAACCCTCCAATGTAAGCGAAGG			

Supplemental Table S5 Primers for serotyping of *G. parasuis*

Serotype	Direction	Targeted gene	Sequence (5′to 3′)	Product size，bp	Temp，°C	Reference
1	Forward	*funB*	TGCATAAAAAATTTTTGAA	1245	49	Jia et al.（8）
	Reverse		TTATATATATTTTACATTTCTAAG			
2	Forward	*funE*	ATGGAAGAAAAAGAATATATC	1032	52	Jia et al.（8）
	Reverse		TTAAAGTTTTGATTTGTCAATG			
3	Forward	*dgdA*	ATGACTAAAAAAATTTTAGTTACAG	1068	52	Jia et al.（8）
	Reverse		TTACTTAATACCTAAGCG			
4	Forward	*gltG*	ATGAATAATAAAGTCTCAATTATAA	753	52	Jia et al.（8）
	Reverse		TTACATATGTTTTACAATTCC			
5	Forward	*funK*	ATGCCAATAGAGATAGC	560	52	Jia et al.（8）
	Reverse		CCTGCCATATTATGA			
6	Forward	*funL*	ATGAGTATTTTTTTTCTAATTG	443	52	Jia et al.（8）
	Reverse		TTCCCTGATCATTGTAGTAACC			
7	Forward	*funQ*	TAGTTGGTATATTATTTTCT	600	52	Jia et al.（8）
	Reverse		AGAATGCATCTGTACCACTAAG			
8	Forward	*scdA*	CAGCAGGTTCTATGGAGTCA	350	49	Jia et al.（8）
	Reverse		CACATTATAACTTTCTTT			
9	Forward	*funV*	GCTCCAATATCAGCAGTA	819	58	Jia et al.（8）
	Reverse		AGAGTAATGAGCATCTCCG			
10	Forward	*funX*	TGATTATTCTACTGCCTTTA	320	55	Jia et al.（8）
	Reverse		CACCTAGCGTAACCCATA			
11	Forward	*actA*	ATGATTATAGGTATTTATGGTGC	657	52	Jia et al.（8）
	Reverse		CTATTTATTTTTTGAAAATTCTC			
12	Forward	*Hypothetical*	ATGGCTCACGATCCGAAAG	508	60	Jia et al.（8）
	Reverse		ATTTCCCTTTCCTAAACGC			
13	Forward	*waaL*	GGCATTAGAGTTTCACCTA	800	60	Jia et al.（8）
	Reverse		TATTAGCATACCCAGCAT			
14	Forward	*funAB*	TGTCTTTGTTACTACTAATTATTG	906	51	Jia et al.（8）
	Reverse		TAGTAACTCCAGATAAAGC			
15	Forward	*funJ*	TTCGCAAGTATAAGGGACT	536	62	Jia et al.（8）
	Reverse		GATGTAGCCATAAAGTCAAT			

Supplemental Table S6 Primers for serotyping of *S. suis*

Serotype	Direction	Targeted gene	Sequence (5′to 3′)	Product size，bp	Temp，°C	Reference
1	Forward	*cps1J*	AATCATGGAATAAAGCGGAGTACAG	550	58	Kerdsin et al.（3）
	Reverse		ACAATTGATACGTCAAAATCCTCACC			
2	Forward	*cps2J*	GATTTGTCGGGAGGGTTACTTG	450	58	Kerdsin et al.（3）
	Reverse		TAAATAATATGCCACTGTAGCGTCTC			
7	Forward	*cps7H*	GATGATTTATGGCACCCGAGTAAGC	150	58	Kerdsin et al.（3）
	Reverse		AGTCACAATTGCTGGTCCTGACACC			
9	Forward	*cps9H*	GGGATGATTGCTCGACAGAT	300	58	Kerdsin et al.（3）
	Reverse		CCGAAGTATCTGGGCTACTG			

Supplemental Table S7 Primers for serotyping of *P. multocida*

Serotype	Direction	Targeted gene	Sequence (5′to 3′)	Product size，bp	Temp，°C	Reference
A	Forward	*hyaD-hyaC*	GATGCCAAAATCGCAGTCAG	1048	56	Peng et al.（4）
	Reverse		TGTTGCCATCATTGTCAGTG			
B	Forward	*bcbD*	CATTTATCCAAGCTCCACC	758	56	Peng et al.（4）
	Reverse		GCCCGAGAGTTTCAATCC			
D	Forward	*dcbF*	TTACAAAAGAAAGACTAGGAGCCC	647	56	Peng et al.（4）
	Reverse		CATCTACCCACTCAACCATATCAG			
E	Forward	*ecbJ*	TCCGCAGAAAATTATTGACTC	512	56	Peng et al.（4）
	Reverse		GCTTGCTGCTTGATTTTGTC			
F	Forward	*fcbD*	TCGGAGAACGCAGAAATCAG	852	56	Peng et al.（4）
	Reverse		TTCCGCCGTCAATTACTCTG			

REFERENCES

1. Zhou L, Jones SCP, Angen Ø, Bossé JT, Nash JHE, Frey J, Zhou R, Chen HC, Kroll JS, Rycroft AN, Langford PR. 2008. Multiplex PCR that can distinguish between immunologically cross-reactive serovar 3, 6, and 8 *Actinobacillus pleuropneumoniae* strains. *J Clin Microbiol* 46:800-803.
2. Howell KJ, Peters SE, Wang J, Hernandez-Garcia J, Weinert LA, Luan SL, Chaudhuri RR, Angen Ø, Aragon V, Williamson SM, Parkhill J, Langford PR, Rycroft AN, Wren BW, Maskell DJ, Tucker AW. 2015. Development of a Multiplex PCR assay for rapid molecular serotyping of *Haemophilus parasuis. J Clin Microbiol* 53:3812-3821.
3. Kerdsin A, Dejsirilert S, Akeda Y, Sekizaki T, Hamada S, Gottschalk M, Oishi K. 2012. Fifteen *Streptococcus suis* serotypes identified by multiplex PCR. *J Med Microbiol* 61:1669-1672.
4. Peng Z, Wang H, Liang W, Chen Y, Tang X, Chen H, Wu B. 2018. A capsule/lipopolysaccharide/MLST genotype D/L6/ST11 of *Pasteurella multocida* is likely to be strongly associated with swine respiratory disease in China. *Arch Microbiol* 200:107-118.
5. Qiu G, Rui Y, Li K, Huang S, Han Z, Wang X, Jiang W, Luo H, Lan Y, Li J. 2017. Detection and phylogenetic analysis of *Mycoplasma hyopneumoniae* from Tibetan pigs in western China. Trop Anim Health Prod 49:1545-1551.
6. Bossé JT, Li Y, Angen Ø, Weinert LA, Chaudhuri RR, Holden MT, Williamson SM, Maskell DJ, Tucker AW, Wren BW, Rycroft AN, Langford PR. 2014. Multiplex PCR assay for unequivocal differentiation of *Actinobacillus pleuropneumoniae* serovars 1 to 3, 5 to 8, 10, and 12. *J Clin Microbiol* 52:2380-2385.
7. Bossé JT, Li Y, Fernandez Crespo R, Lacouture S, Gottschalk M, Sárközi R, Fodor L, Casas Amoribieta M, Angen Ø, Nedbalcova K, Holden MTG, Maskell DJ, Tucker AW, Wren BW, Rycroft AN, Langford PR. 2018. Comparative sequence analysis of the capsular polysaccharide loci of *Actinobacillus pleuropneumoniae* serovars 1-18, and development of two multiplex PCRs for comprehensive capsule typing. *Vet Microbiol* 220:83-89.
8. Jia A, Zhou R, Fan H, Yang K, Zhang J, Xu Y, Wang G, Liao M. 2017. Development of serotype-specific PCR assays for typing of *Haemophilus parasuis* isolates circulating in Southern China. *J Clin Microbiol* 55:3249-3257.
